# Supplementary material for: Robust continuous in vitro culture of the Plasmodium cynomolgi erythrocytic stages
Source: Nat Commun. 2019 Aug 12;10:3635. doi: 10.1038/s41467-019-11332-4 (PMC6690977; doi:10.1038/s41467-019-11332-4)
Supplement: Supplementary file 1 — Supplementary Information [file 41467_2019_11332_MOESM1_ESM.pdf]

SUPPLEMENTARY INFORMATION

Robust continuous *in vitro* culture of the *Plasmodium cynomolgi* erythrocytic stages

Adeline C.Y. Chua[1,2,3§], Jessica Jie Ying Ong[2,3§], Benoit Malleret[1,4], Rossarin Suwanarusk[1,2], Varakorn Kosaisavee[4,5], Anne-Marie Zeeman[6], Caitlin A. Cooper[7], Kevin S.W. Tan[4], Rou Zhang[4], Bee Huat Tan[3], Siti Nurdiana Abas[3], Andy Yip[3], Anne Elliot[7], Chester J. Joyner[8,10], Jee Sun Cho[4], Kate Breyer[11], Szczepan Baran[11], Amber Lange[11,] Steven P. Maher[7], François Nosten[12,13], Christophe Bodenreider[3], Bryan K. S. Yeung[3], Dominique Mazier[14,15], Mary R. Galinski[9,10], Nathalie Dereuddre-Bosquet[15], Roger Le Grand[15], Clemens H. M. Kocken[6], Laurent Rénia[1,4], Dennis E. Kyle[7], Thierry T. Diagana[3], Georges Snounou[14,15,16], Bruce Russell[2,4*], Pablo Bifani[1, 3,4,17*]

[1] Singapore Immunology Network, A*STAR, 138648 Singapore

[2] Department of Microbiology and Immunology, University of Otago, Dunedin 9054, New Zealand

[3] Novartis Institute for Tropical Diseases, 138670 Singapore

[4] Department of Microbiology and Immunology, Yong Loo Lin School of Medicine, National University of Singapore, 119077 Singapore

[5] Department of Parasitology and Entomology, Faculty of Public Health, Mahidol University, Bangkok, Thailand

[6] Department of Parasitology, Biomedical Primate Research Centre, Rijswijk, The Netherlands

[7] Center for Tropical and Emerging Global Diseases, University of Georgia, Athens, GA, USA

[8] Division of Pulmonary, Allergy, Critical Care & Sleep Medicine, Emory University, Atlanta, GA USA

[9] Division of Infectious Diseases, Department of Medicine, Emory University, Atlanta, GA, USA

[10] Emory Vaccine Center, Emory University, Atlanta, GA, USA

[11] Laboratory Animal Services, Scientific Operations, Novartis Institutes for Biomedical Research, East Hanover, NJ 07936-1080, USA

[12] Shoklo Malaria Research Unit, Mahidol-Oxford Tropical Medicine Research Unit, Faculty of Tropical Medicine, Mahidol University, Mae Sot, Thailand

[13] Centre for Tropical Medicine and Global Health, Nuffield Department of Medicine Research Building, , University of Oxford Old Road Campus, OxfordUnited Kingdom

[14] Sorbonne Universités, Université Pierre et Marie Curie, UMR S945 Paris, France

[15] Centre d'Immunologie et de Maladies Infectieuses-Paris, INSERM U1135, Centre National de la Recherche Scientifique, Paris, France

[16] CEA-Université Paris Sud 11-INSERM U1184, Immunology of Viral Infections and Autoimmune Diseases (IMVA), IDMIT Department, IBJF, DRF, Fontenay-aux-Roses, France

[17] Faculty of Infectious and Tropical Diseases, London School of Hygiene & Tropical Medicine, London, London WC1E 7HT, United Kingdom

§Equal contributions

*Correspondence: BR: b.russell@otago.ac.nz or PB: micpb@nus.edu.sg

Supplementary Figure 1

Gene	Strain	Forward primer (5' → 3')	Reverse primer (5' → 3')	Product size (bp)
rbp1b	Berok (K4)	CGATAATTTGACAGCGGCGG	GCCCTATATCGGGTTCTGCC	875
rbp2a	B, M	AATTGTTCAGCACACCAGAAGG	ACAAACACCCAACCCCAAAATC	149
rbp2b	B, M	TGGAAATAAACGCACTTTGGACG	GGGGAGCCTGTTTTGGTGAG	668
rpb2b	Berok (K4)	CGCACTTTGGACGATTTTATGC	GGGTACTTTTCGAGGGAGCC	670

Gene	M	B	Berok (K4)
rbp1b	-	-	+
rbp2a	+	+	-
rbp2b	+	+	+

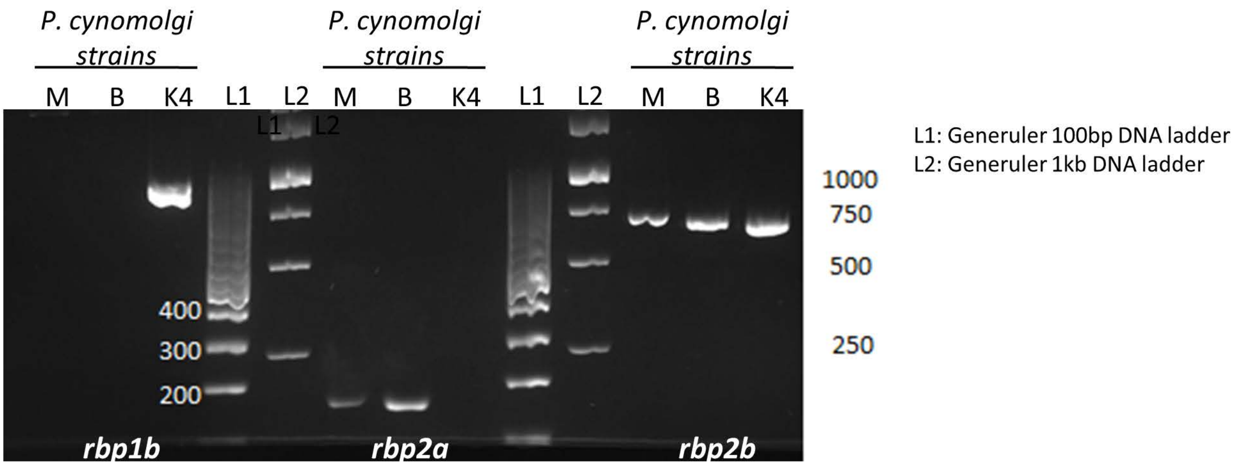

Supplementary Figure 1. Genotyping of the *reticulocyte binding protein* genes (*rbp1* and *rbp2*) in the continuous culture of *P. cynomolgi* Berok K4 strain.

Supplementary Figure 2

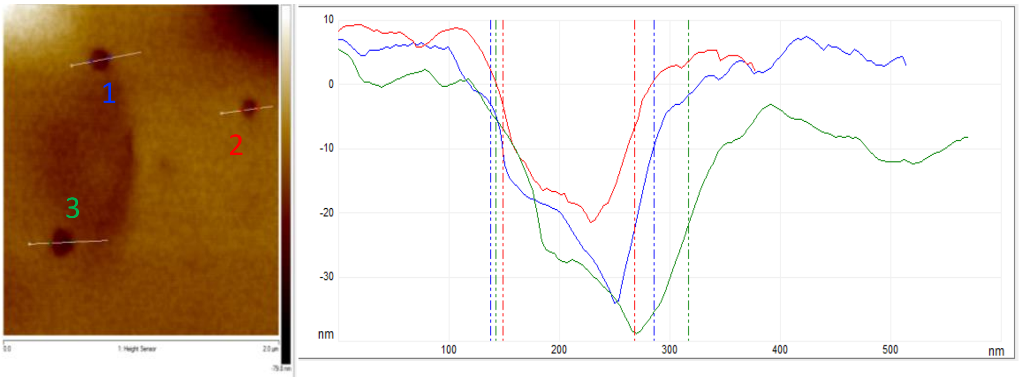

Pair	Horizontal Distance	Vertical Distance	Surface Distance	Angle	Rmax	Rz	Rz Count	Rms	Ra (Frequency Cutoff)	Frequency Cutoff	Radius	Radius Sigma
1	0.147 (µm)	-6.738 (nm)	0.161 (µm)	-2.630 (º)	31.867 (nm)	0.000 (nm)	0.000	3.679 (nm)	16.600 (nm)	1.911 (µm)	0.116 (µm)	0.005 (µm)
2	0.118 (µm)	-3.252 (nm)	0.125 (µm)	-1.576 (º)	15.453 (nm)	0.000 (nm)	0.000	0.967 (nm)	13.305 (nm)	2.590 (µm)	0.131 (µm)	0.001 (µm)
3	0.172 (µm)	-15.879 (nm)	0.182 (µm)	-5.265 (º)	31.211 (nm)	0.000 (nm)	0.000	4.683 (nm)	15.748 (nm)	0.000 (µm)	0.187 (µm)	0.003 (µm)

Supplementary Figure 2. Measurement of the diameter of the caveolae structures observed on the surface of the infected red blood cells of the continuous culture of *P. cynomolgi* Berok K4 strain by atomic force microscopy.

Supplementary Figure 3

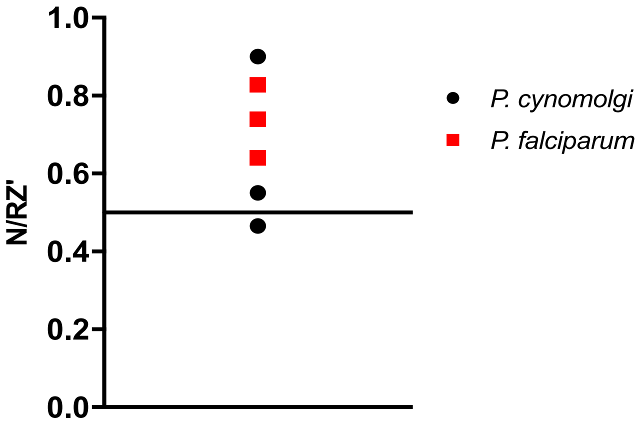

Supplementary Figure 3. The robustness (Z' value) of the adapted SYBR green I proliferation assay for the continuous culture of *P. cynomolgi* Berok K4 strain was similar to the SYBR green I proliferation assay routinely used for *P. falciparum.*

Supplementary Figure 4

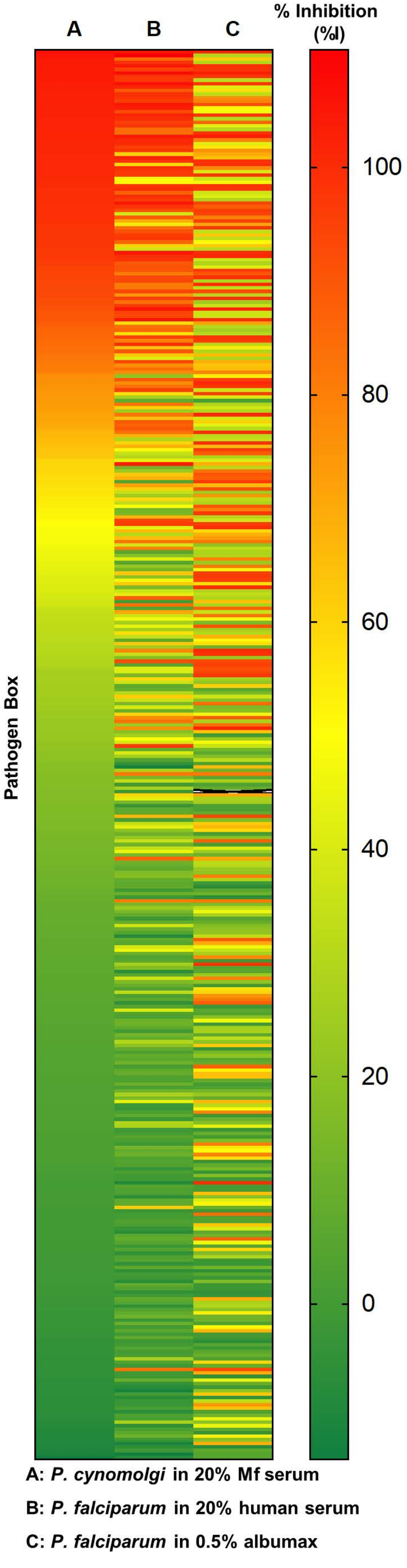

Supplementary Figure 4. Heat map showing percentage of inhibition across 400 compounds from the Pathogen Box. Antimalarial reference compounds from the Pathogen Box gave specific activity against *P. falciparum* and *P. cynomolgi* while reference compounds against different pathogens were inactive.
