# Supplementary material for: Robust continuous in vitro culture of the Plasmodium cynomolgi erythrocytic stages
Source: Nat Commun. 2019 Aug 12;10:3635. doi: 10.1038/s41467-019-11332-4 (PMC6690977; doi:10.1038/s41467-019-11332-4)
Supplement: Supplementary file 3 — Reporting Summary [file 41467_2019_11332_MOESM3_ESM.pdf]

nature research

Corresponding author(s): Pablo Bifani

Last updated by author(s): Jun 18, 2019

# Reporting Summary

Nature Research wishes to improve the reproducibility of the work that we publish. This form provides structure for consistency and transparency in reporting. For further information on Nature Research policies, see Authors & Referees and the Editorial Policy Checklist .

## Statistics

For all statistical analyses, confirm that the following items are present in the figure legend, table legend, main text, or Methods section.

| n/a | Confirmed | |
|---|---|---|
| ☐ | ☒ | The exact sample size ($n$) for each experimental group/condition, given as a discrete number and unit of measurement |
| ☐ | ☒ | A statement on whether measurements were taken from distinct samples or whether the same sample was measured repeatedly |
| ☐ | ☒ | The statistical test(s) used AND whether they are one- or two-sided<br>*Only common tests should be described solely by name; describe more complex techniques in the Methods section.* |
| ☒ | ☐ | A description of all covariates tested |
| ☐ | ☒ | A description of any assumptions or corrections, such as tests of normality and adjustment for multiple comparisons |
| ☐ | ☒ | A full description of the statistical parameters including central tendency (e.g. means) or other basic estimates (e.g. regression coefficient) AND variation (e.g. standard deviation) or associated estimates of uncertainty (e.g. confidence intervals) |
| ☐ | ☒ | For null hypothesis testing, the test statistic (e.g. $F$, $t$, $r$) with confidence intervals, effect sizes, degrees of freedom and $P$ value noted<br>*Give P values as exact values whenever suitable.* |
| ☒ | ☐ | For Bayesian analysis, information on the choice of priors and Markov chain Monte Carlo settings |
| ☒ | ☐ | For hierarchical and complex designs, identification of the appropriate level for tests and full reporting of outcomes |
| ☒ | ☐ | Estimates of effect sizes (e.g. Cohen's $d$, Pearson's $r$), indicating how they were calculated |

*Our web collection on statistics for biologists contains articles on many of the points above.*

## Software and code

Policy information about availability of computer code

| | |
|---|---|
| Data collection | Data for Figure 1d and Figure 3g were collected using Olympus cellSens software.<br>Data for Figure 2b were collected using Operetta high-content imaging system.<br>Data for Figure 3a were collected using JEOL software, data for Figure 3c were collected using Nanoscope 5.3 software and data for Figure 3e were collected using IDEAS software 4.0.<br>Data for Figure 5a were collected using FlowJo 10.1 software. |
| Data analysis | Graph Pad Prism 7 was used for data analysis. |

For manuscripts utilizing custom algorithms or software that are central to the research but not yet described in published literature, software must be made available to editors/reviewers. We strongly encourage code deposition in a community repository (e.g. GitHub). See the Nature Research guidelines for submitting code & software for further information.

## Data

Policy information about availability of data

All manuscripts must include a data availability statement. This statement should provide the following information, where applicable:

- Accession codes, unique identifiers, or web links for publicly available datasets
- A list of figures that have associated raw data
- A description of any restrictions on data availability

The source data underlying Figures 1b, 1c, 2c, 2d, 3a-h, 4a-c, 5b-d, Supplementary Figures 1 and 4 are provided in the Source Data file.

# Field-specific reporting

Please select the one below that is the best fit for your research. If you are not sure, read the appropriate sections before making your selection.

☒ Life sciences ☐ Behavioural & social sciences ☐ Ecological, evolutionary & environmental sciences

For a reference copy of the document with all sections, see nature.com/documents/nr-reporting-summary-flat.pdf

# Life sciences study design

All studies must disclose on these points even when the disclosure is negative.

| | |
|---|---|
| Sample size | No sample size calculations were performed.<br>Sample sizes were determined based on our extensive experience in this type of experimental approach. |
| Data exclusions | There were no data exclusions from the analyses. |
| Replication | All of the replication attempts were successful and all the attempts were included in the analyses. |
| Randomization | Randomization was not necessary for this experimental design. |
| Blinding | The compounds tested as per Fig. 4b and 4c were single blinded. |

# Reporting for specific materials, systems and methods

We require information from authors about some types of materials, experimental systems and methods used in many studies. Here, indicate whether each material, system or method listed is relevant to your study. If you are not sure if a list item applies to your research, read the appropriate section before selecting a response.

## Materials & experimental systems

| n/a | Involved in the study | |
|---|---|---|
| ☐ | ☒ | Antibodies |
| ☒ | ☐ | Eukaryotic cell lines |
| ☒ | ☐ | Palaeontology |
| ☐ | ☒ | Animals and other organisms |
| ☒ | ☐ | Human research participants |
| ☒ | ☐ | Clinical data |

## Methods

| n/a | Involved in the study | |
|---|---|---|
| ☒ | ☐ | ChIP-seq |
| ☐ | ☒ | Flow cytometry |
| ☒ | ☐ | MRI-based neuroimaging |

## Antibodies

| | |
|---|---|
| Antibodies used | Anti-PcHSP antibodies (Zeeman et al. 2014 AAC (58):1586) and goat-anti-rabbit Alexa568 red secondary antibodies (Invitrogen A11011) were used for Figure 2b. |
| Validation | The primary anti-PcHSP antibodies were optimized and validated for use in the liver stage assay as stated in Zeeman et al. (2014) AAC 58:1586. |

## Animals and other organisms

Policy information about studies involving animals; ARRIVE guidelines recommended for reporting animal research

| | |
|---|---|
| Laboratory animals | Macaca fascicularis and Macaca mulatta monkeys were used in this study. |
| Wild animals | No wild animals were used in this study. |
| Field-collected samples | No samples collected from the field were used in this study. |
| Ethics oversight | All animals were housed in accordance with the Guide for the Care and Use of Laboratory Animals and the Association for the Assessment and Accreditation of Laboratory Animal Care (AAALAC) Standards.<br>All studies were approved by the Novartis Ethical Review Council and Novartis Institutional Animal Care and Use Committees prior (IACUC), SingHealth Experimental Medicine Center (IACUC), European directive 2010/63/EU and with the 'Standard for humane care and use of Laboratory Animals by Foreign institutions' identification number A5539-01, provided by the Department of Health and Human Services of the USA National Institutes of Health (NIH) for studies carried out in BPRC and OXTREC 45-09 (University of Oxford, Centre for Clinical Vaccinology and Tropical Medicine, UK) and MUTM 2008-215 from the |

Ethics committee of Faculty of Tropical Medicine, Mahidol University for studies carried out in SMRU.

Note that full information on the approval of the study protocol must also be provided in the manuscript.

# Flow Cytometry

## Plots

Confirm that:

- [x] The axis labels state the marker and fluorochrome used (e.g. CD4-FITC).
- [x] The axis scales are clearly visible. Include numbers along axes only for bottom left plot of group (a 'group' is an analysis of identical markers).
- [x] All plots are contour plots with outliers or pseudocolor plots.
- [x] A numerical value for number of cells or percentage (with statistics) is provided.

## Methodology

| | |
|---|---|
| Sample preparation | The respective treated/ untreated P. cynomolgi Berok K4 strain samples in Fig. 5a were mixed well and an aliquot was stained with Hoechst and dihydroethidium (DHE). |
| Instrument | The data were collected with the BD Accuri C6. |
| Software | The FlowJo 10.1 software was used for data analysis. |
| Cell population abundance | In the control group, the abundance of the target cell was approximately 0.1% and 0.2% (please see Fig. 5a). |
| Gating strategy | The well cited and widely used gating strategy inthe malaria field as described in Malleret et al. 2011. Science Reports (1): 118 was used in this study.<br>Briefly, 100,000 events were acquired and they were first gated by FSC-SSC before duplets were excluded. |

- [ ] Tick this box to confirm that a figure exemplifying the gating strategy is provided in the Supplementary Information.
